# Lactic Acid Bacteria Strains Used as Starters for Kimchi Fermentation Protect the Disruption of Tight Junctions in the Caco-2 Cell Monolayer Model

**DOI:** 10.4014/jmb.2209.09026

**Published:** 2022-11-17

**Authors:** Jin Yong Kang, Moeun Lee, Jung Hee Song, Eun Ji Choi, Da un Kim, Seul Ki Lim, Namhee Kim, Ji Yoon Chang

**Affiliations:** Research and Development Division, World Institute of Kimchi, Gwangju 61755, Republic of Korea

**Keywords:** Kimchi, fermentation, lactic acid bacteria, tight junction, Caco-2

## Abstract

In this study, we investigated the effect of lactic acid bacteria (LAB) strains used as starters for kimchi fermentation, namely *Lactococcus lactis* WiKim0124, *Companilactobacillus allii* WiKim39, *Leuconostoc mesenteroides* WiKim0121 *Leuconostoc mesenteroides* WiKim33, and *Leuconostoc mesenteroides* WiKim32, on the intestinal epithelial tight junctions (TJs). These LAB strains were not cytotoxic to Caco-2 cells at 500 μg/ml concentration. In addition, hydrogen peroxide (H_2_O_2_) decreased Caco-2 viability, but the LAB strains protected the cells against H_2_O_2_-induced cytotoxicity. We also found that lipopolysaccharide (LPS) promoted Caco-2 proliferation; however, no specific changes were observed upon treatment with LAB strains and LPS. Our evaluation of the permeability in the Caco-2 monolayer model confirmed its increase by both LPS and H_2_O_2_. The LAB strains inhibited the increase in permeability by protecting TJs, which we evaluated by measuring TJ proteins such as zonula occludens-1 and occludin, and analyzing them by western blotting and immunofluorescence staining. Our findings show that LAB strains used for kimchi fermentation can suppress the increase in intestinal permeability due to LPS and H_2_O_2_ by protecting TJs. Therefore, these results suggest the possibility of enhancing the functionality of kimchi through its fermentation using functional LAB strains.

## Introduction

Kimchi, a traditional fermented food in Korea, is attracting attention worldwide. Numerous microorganisms are involved in kimchi fermentation, producing various metabolites. During fermentation, putrefactive bacteria are suppressed and lactic acid bacteria (LAB) become dominant [1]. Among the LAB, the genera *Leuconostoc*, *Weissella*, and *Lactobacillus* are believed to be the key players in kimchi fermentation [2]. Controlling the process of kimchi fermentation is challenging due to its numerous variables. Accordingly, kimchi produced through natural fermentation often differs in terms of quality. The use of starter cultures has been considered an alternative to address these problems [3]. A starter culture is a microbial preparation of large numbers of cells of at least one microorganism added to the raw material to produce fermented foods, such as yogurt, kefir, sauerkraut, tempeh, and kimchi by accelerating and steering the fermentation process. LAB strains occupy a central role in these processes as they enhance the end product’s microbial safety, shelf life, and texture [4]. Therefore, various studies are being conducted to control kimchi fermentation using kimchi starter cultures and to further develop the kimchi manufacturing industry [3].

Kimchi starters characterize by adapting to the unique environment of kimchi fermentation, which depends on low temperature, low pH, and presence of NaCl [5, 6]. Researchers are currently exploring functional starter cultures, which, along with their function as simple starters for fermentation control, also contribute to food safety and offer one or more organoleptic, technological, nutritional, or health advantages for the food fermentation industry [7].

Tight junctions (TJs) are protein complexes, such as occludin and various claudins, as well as cytoplasmic plaque proteins, such as zonula occludens-1 (ZO-1) and zonula occludens-2 (ZO-2), which maintain epithelial barrier integrity [8]. ZO-1 and occludin play a pivotal role in tissue differentiation and organogenesis, and are considered key molecules in cell-cell interaction [9]. TJs regulate the permeability of the intestinal barrier and act as barriers between epithelial and endothelial cells [10]. Disruption of the intestinal epithelial barrier can lead to various diseases [11]. Several studies have demonstrated that some LAB promote the significant upregulation and relocalization of interepithelial TJ proteins [12].

Therefore, this study was conducted to develop kimchi starter cultures using a functional starter by confirming whether the strains used can positively affect TJs.

## Materials and Methods

### Kimchi Starter Cultures

*Lactococcus lactis* WiKim0124 (WiKim0124), *Companilactobacillus allii* WiKim39 (WiKim39), *Leuconostoc mesenteroides* WiKim0121 (WiKim0121), *Leuconostoc mesenteroides* WiKim33 (WiKim33), and *Leuconostoc mesenteroides* WiKim32 (WiKim32) were isolated from kimchi [13, 14]. LAB strains for kimchi fermentation were cultured in deMan, Rogosa, and Sharpe agar media (Difco Laboratories Inc, USA) at 30°C and collected by centrifugation (4,000 ×*g*, 10 min, 4°C). Subsequently, they were dissolved in Dulbecco’s phosphate-buffered saline (DPBS) and homogenized using a Bandelin ultrasonic homogenizer (Bandelin Electronic GmbH & Co. KG, Germany). Supernatants were obtained by centrifugation (13,000 ×*g*, 10 min, 4°C) and lyophilized using a vacuum freeze dryer. The obtained lyophilizates dissolved in DPBS were used in the experiments.

### Caco-2 Cell Culture and Monolayer Model

Human intestinal epithelial Caco-2 cells (ATCC TIB-71, USA) were incubated (37°C in a 5% CO_2_ atmosphere) in Dulbecco’s modified Eagle’s medium containing 20% fetal bovine serum and 1% penicillin/streptomycin. To form a Caco-2 cell monolayer model, Caco-2 cells were grown to confluence and incubated for 21 days, with the medium being changed once every two days.

### Cell Viability and Proliferation

Sample cytotoxicity was evaluated using the XTT assay, which measures cell viability [15]. The Caco-2 cells were seeded in a 96-well plate at 1×10^4^/well and incubated for 24 h. After LAB strain treatment, the cells were incubated for another 24 h. Then, the medium was removed by suction, and the sample was washed with DPBS and treated with XTT solution for 3 h. The supernatants were obtained by centrifugation (4,000 ×*g*, 10 min, 4°C), and the absorbance was measured at 450 nm. The protective effect against lipopolysaccharide (LPS)- or hydrogen peroxide (H_2_O_2_)-induced cell cytotoxicity was evaluated using the XTT assay. After treating Caco-2 cells with the LAB strains, the cells were exposed to LPS (100 μg/ml) for 24 h and H_2_O_2_ (500 μM) for 5 h. Cell viability was confirmed using the XTT assay.

### Evaluation of Caco-2 Monolayer Model Permeability

Caco-2 cells were seeded onto collagen-coated Transwell filters with a pore size of 0.8 mm (Corning Incorporated, USA) to evaluate intestinal permeability. After the Caco-2 monolayer was formed, the LAB strains were added and incubated with either LPS (100 μg/ml for 24 h) or H_2_O_2_ (500 μM for 5 h). Then, the medium was replaced with a medium with 1 mg/ml 40 kDa fluorescein isothiocyanate (FITC)-labeled dextran (Sigma-Aldrich, USA) in the apical chamber of the Transwell. After 6 h of incubation, the media in the basolateral chamber were collected, and the fluorescence was measured at 485 nm excitation and 535 nm emission wavelengths.

### Immunofluorescence Staining

The LAB strains were incubated with either LPS (100 μg/ml for 24 h) or H_2_O_2_ (500 μM for 5 h) on the monolayer. The cells were fixed with 4% paraformaldehyde for 15 min and blocked with 3% bovine serum albumin for 1 h. After the cells were rinsed with DPBS, primary antibodies, such as ZO-1 (1:100, 61-7300, Thermo Fisher Scientific, USA) and occludin (1:100, 42-2400, Thermo Fisher Scientific), were added and incubated at 4°C overnight. After rinsing with phosphate-buffered saline with Tween 20 (PBST) three times, the samples were incubated with Alexa Fluor 488-labeled secondary antibodies (1:1000) for 1 h at room temperature. Cell nuclei were stained by DAPI. Finally, the cells were observed under a fluorescence microscope after dropping the FluoromountTM Aqueous Mounting Medium (Sigma-Aldrich).

### Western Blot Assay

The cells were harvested using RIPA buffer containing a 1% protease inhibitor. Then, they were homogenized using Bandelin ultrasonic homogenizer, and the homogenates were centrifuged at 13,000 ×*g* for 10 min at 4°C. The supernatants were used in the experiments. Proteins were separated using sodium dodecyl sulfate-polyacrylamide gel electrophoresis and electro-transferred to a polyvinylidene difluoride membrane. The membranes were incubated with primary antibodies (ZO-1 and occludin) overnight at 4°C. After rinsing with PBST three times, the membrane was incubated with a secondary antibody (1:1000) solution for 1 h at room temperature. The membrane was then treated with a chemiluminescence reagent and rinsed with PBST three times. The bands were observed using Gel Doc XR+ equipment (Bio-Rad Laboratories, Inc., USA).

### Statistical Analysis

Data are represented as mean ± SD. Tukey's honest significant difference (HSD) test was carried out using the “Agricolae” package of the R software (v3.3.2; https://www.r-project.org/) for group comparisons.

## Results and Discussion

### Effect of LAB Strains on Caco-2 Cells

**Fig. 1:**
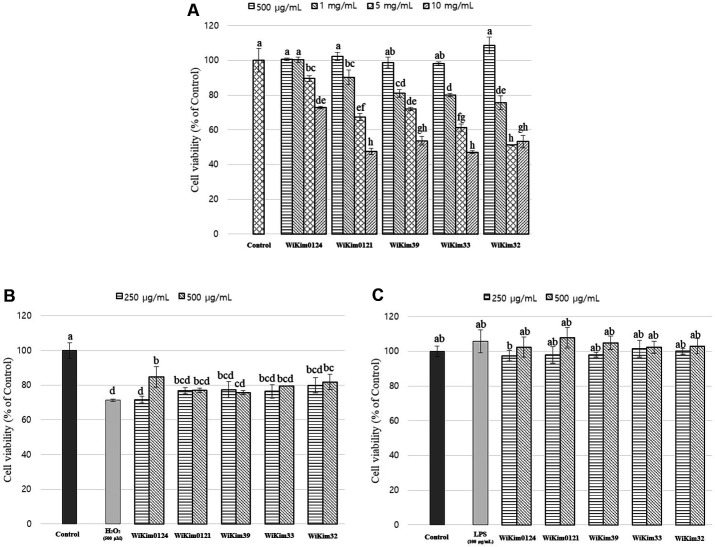
The effect of LAB strains on Caco-2 cell viability. (**A**) Toxicological evaluation of LAB strains through XTT assay. (**B**) Effect of LAB strains on H_2_O_2_ treatment to the cell. (**C**) Effect of LAB strains on LPS treatment to the cell. Results are indicated as mean ± SD (*n* = 3). Different letters represent statistical differences (*p* < 0.05, ANOVA, Tukey-HSD), and alphabetical order indicates value size of result.

The LAB strains, except for WiKim0124, posed cytotoxicity to Caco-2 cells at a concentration of 1 mg/ml, and no other LAB strain showed cytotoxicity at 500 μg/ml concentration (Fig. 1A). Treatment with LPS and H_2_O_2_ induced Caco-2 cell proliferation and cytotoxicity, respectively (Figs. 1B and 1C). According to Lin *et al*. (2015), LPS promotes cellular growth via c-Src upregulation in Caco-2 cells [16]. Accordingly, the tendency to increase cell viability was confirmed in the LPS-treated group, and there was no notable change in the treatment of LAB strains to Caco-2 cells treated with LPS (Fig. 1C). However, treatment with H_2_O_2_ reduced cell viability by approximately 29% (Fig. 1B). H_2_O_2_ rapidly kills any cell type through the oxidation of proteins, membrane lipids, and DNA by producing highly reactive hydroxyl radicals [17]. However, this oxidative damage can be inhibited by antioxidant activity.

Increased intake of exogenous antioxidants would alleviate the damage caused by oxidative stress by inhibiting the initiation or propagation of oxidative chain reaction, acting as free radical scavengers, quenchers of singlet oxygen, and reducing agents [18]. Natural antioxidants are widely distributed in food and medicinal plants [19]. The biological effects of probiotics are strain-specific, and although the success of one strain cannot be extrapolated to another strain, several studies have confirmed the antioxidant properties of various LAB [20–23]. In addition, WiKim0124 and WiKim39 strains were reported in a previous study to have antioxidant activity [24].

### Effect of LAB Strains on Caco-2 Monolayer Permeability

**Fig. 2:**
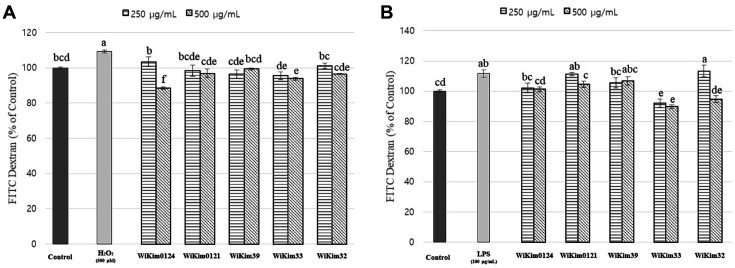
The effect of LAB strains on the permeability in the Caco-2 monolayer model. (**A**) Effect of LAB strains on H_2_O_2_-induced permeability change of Caco-2 monolayer model. (**B**) Effect of LAB strains on LPS-induced permeability change of Caco-2 monolayer model. Results are indicated as mean ± SD (*n* = 3). Different letters represent statistical differences (*p* < 0.05, ANOVA, Tukey-HSD), and alphabetical order indicates value size of result.

Altered intestinal permeability influences many pathological conditions. The critical roles of intestinal permeability and barrier in health have been consistently confirmed [25]. In our experiments, both LPS and H_2_O_2_ increased permeability (Fig. 2). Although the mechanisms may differ, LPS and H_2_O_2_ were previously reported to increase permeability [26, 27]. WiKim0124, at a treatment concentration of 500 μg/ml, was most effective in suppressing the increased permeability due to H_2_O_2_. These results are presumed to be related to the relatively higher protective effect of WiKim0124 against H_2_O_2_-induced cytotoxicity (Fig. 1B). H_2_O_2_ induces oxidative stress, resulting in the loss of membrane integrity, increased permeability, and defective membrane transport mechanisms through oxidative degradation of membrane lipids [28]. Our findings indicate the antioxidant activity of WiKim0124 in mitigating H_2_O_2_ toxicity.

WiKim33 and WiKim32 were relatively effective in inhibiting the LPS-induced increase in permeability (Fig. 2B). Initially, LPS was not toxic to cells but promoted cell differentiation (Fig. 1C); however, LPS significantly increased permeability (Fig. 2B). Permeability is controlled by endothelial cell substructural components reacting with a multitude of vasoactive cytokines, signal modulators, and growth factors. LPS induces the loss of the junction barrier through the activation of protein tyrosine kinases (PTKs) and caspase cleavage reactions [29]. In addition, LPS causes an increase in intestinal permeability via an intracellular mechanism involving TLR-4-dependent upregulation of CD14 membrane expression [30]. Here, the LAB strains effectively suppressed the increased LPS-induced permeability, and although the mechanism underlying the efficacy of LAB strains is yet to be elucidated, various LAB strains are regularly being reported to improve intestinal permeability effectively in in vivo and in vitro models [31-33]. In addition, probiotics reduce intestinal permeability in human patients [34]. More detailed research is needed, but nevertheless, our study demonstrates that WiKim0124 is effective in inhibiting the disruption of TJs due to oxidative stress, and further, that WiKim32 and WiKim33 are effective in inhibiting the disruption of TJs due to LPS.

### Effect of LAB Strains on TJ Proteins

**Fig. 3:**
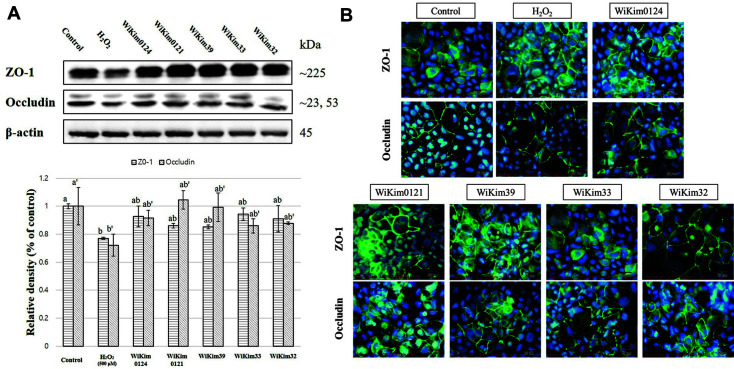
The effect of LAB strains at 500 μg/ml on tight junction proteins in H_2_O_2_-treated Caco-2 monolayer model. (**A**) Relative density of TJ proteins through western blot assay. (**B**) Representative image of TJ proteins through immunofluorescent staining. Results are indicated as mean ± SD (*n* = 3). Different letters represent statistical differences (*p* < 0.05, ANOVA, Tukey-HSD), and alphabetical order indicates value size of result.

**Fig. 4:**
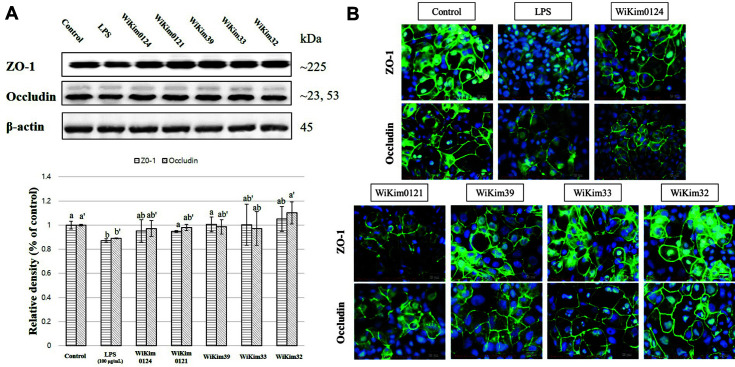
The effect of LAB strains at 500 μg/ml on tight junction proteins in the LPS-treated Caco-2 monolayer model. (**A**) Relative density of TJ proteins through western blot assay. (**B**) Representative image of TJ proteins through immunofluorescent staining. Results are indicated as mean ± SD (*n* = 3). Different letters represent statistical differences (*p* < 0.05, ANOVA, Tukey-HSD), and alphabetical order indicates value size of result.

The human intestinal epithelium is formed by a single layer of epithelial cells. The space between these cells is sealed by TJs, which regulate the permeability of the intestinal barrier and act as a barrier between epithelial and endothelial cells [8, 35]. A decrease in TJ proteins, such as ZO-1 and occludin, was confirmed in the LPS- and H_2_O_2_-treated groups with increased permeability (Figs. 3A and 4A). As a result of observing ZO-1 and occludin proteins through immunofluorescence staining, the destruction of TJ proteins was also observed in the LPS- and H_2_O_2_-treated groups (Figs. 3B and 4B). H_2_O_2_ causes a loss of occludin and ZO-1 junctional localization, with subsequent punctuated staining at TJs, and causes stress fiber formation [36]. H_2_O_2_-induced decrease in electrical resistance and increase in inulin permeability are associated with the dephosphorylation of occludin on threonine residues [37]. In addition, claudins and occludins are often targeted and misplaced by viruses [38, 39], bacteria [40, 41], and inflammatory cytokines [41, 42]. Furthermore, LPS reduces the expression of TJ proteins such as occludin and ZO-1 [43].

LAB strains inhibited the reduction in TJ proteins caused by LPS or H_2_O_2_ treatment (Figs. 3A and 4A), supporting the hypothesis that intestinal permeability was improved in the group of LAB strains treated with LPS or H_2_O_2_. However, studies on bioactive substances or related mechanisms of these effects of LAB strains remain lacking. Studies have only reported that some LAB strains increase the expression of TJ proteins. Specific *Lactobacillus* species abate barrier disruption by upregulating TJ protein expression, while *L. acidophilus* and *L. plantarum* have increased occludin protein expression in in vivo and in vitro models, respectively [31, 32]. The beneficial effects of probiotics on intestinal barrier function are at least partially mediated through organismal stimulation of toll-like receptors (TLRs), particularly TLR2, and sequential changes in TJ protein expression and localization [12]. Although it is necessary to confirm the exact mechanism of improvement of each strain for these TJ changes, this study is meaningful in confirming that strains used for kimchi fermentation positively affect intestinal permeability.

In conclusion, LAB strains used for kimchi fermentation effectively alleviate the disruption of intestinal permeability in the Caco-2 cell monolayer model. These LAB strains had a protective effect against Caco-2 cytotoxicity and increased permeability caused by H_2_O_2_. In addition, although LPS was not toxic to cells, it increased permeability. LAB strains inhibited the LPS-induced permeability by inhibiting the reduction of TJ proteins such as ZO-1 and occludin caused by LPS and H_2_O_2_. Further and more diverse studies are necessary to investigate not only the mechanisms behind the functionality of LAB strains, but also to identify other strains with highly beneficial activities. Although our study did not identify such a strain, our findings should stimulate the future development of the kimchi starter culture industry.
